# Identification of potential diagnostic markers and molecular mechanisms of asthma and ulcerative colitis based on bioinformatics and machine learning

**DOI:** 10.3389/fmolb.2025.1554304

**Published:** 2025-05-15

**Authors:** Chenxuyu Zhang, Zheng Luo, Liang Ji

**Affiliations:** ^1^ Mianyang Hospital of Traditional Chinese Medicine, Mianyang, China; ^2^ Clinical Medical College, Chengdu University of Traditional Chinese Medicine, Chengdu, China; ^3^ Chengdu University of Traditional Chinese Medicine, Chengdu, China

**Keywords:** bioinformatics analysis, machine learning, ulcerative colitis, asthma, immune infiltration, hub genes, molecular dynamics simulation

## Abstract

**Backgrounds:**

Asthma and ulcerative colitis (UC) are chronic inflammatory diseases linked through the “gut-lung axis,” but their shared mechanisms remain unclear. This study aims to identify common biomarkers and pathways between asthma and UC using bioinformatics.

**Methods:**

Gene expression data for asthma and UC were retrieved from the GEO database, and differentially expressed genes (DEGs) were analyzed. Weighted Gene Coexpression Network Analysis (WGCNA) identified UC-associated gene modules. Shared genes between asthma and UC were derived by intersecting DEGs with UC-associated modules, followed by functional enrichment and protein-protein interaction (PPI) analysis. Machine learning identified hub genes, validated through external datasets using ROC curves, nomograms, and boxplots. Gene Set Enrichment Analysis (GSEA) explored pathway alterations, while immune infiltration patterns were analyzed using the CIBERSORT algorithm. Molecular docking (MD) was performed to predict therapeutic compounds, followed by molecular dynamics simulations on the top-ranked docked complex to assess its binding stability.

**Results:**

A total of 41 shared genes were identified, linked to inflammatory and immune pathways, including TNF, IL-17, and chemokine signaling. Four key hub genes—NOS2, TCN1, CHI3L1, and TIMP1—were validated as diagnostic biomarkers. Immune infiltration analysis showed strong correlations with multiple immune cells. Molecular docking identified several potential therapeutic compounds, with PD 98059, beclomethasone, and isoproterenol validated as promising candidates. The stability of the TIMP1-Beclomethasone complex was determined through molecular dynamics simulations.

**Conclusion:**

This study highlights NOS2, TCN1, CHI3L1, and TIMP1 as potential biomarkers and therapeutic targets for asthma and UC, providing insights into shared mechanisms and new strategies for diagnosis and treatment.

## Graphical Abstract

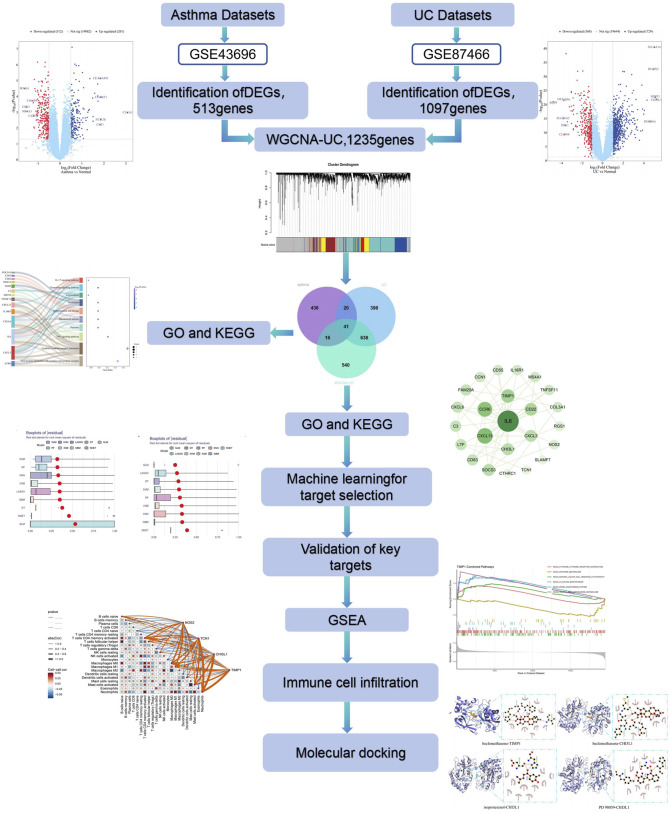

## 1 Introduction

Asthma is a chronic inflammatory airway disease marked by symptoms such as wheezing, nocturnal cough, shortness of breath, chest tightness, and reduced expiratory volume (Lin et al., 2019). It results from the interaction between genetic and environmental factors. The disease is characterized by immune dysregulation, chronic inflammation, tissue remodelling, and heightened tissue sensitivity, all of which vary dynamically across individuals (Agache and Akdis, 2019). Asthma affects around 10% of children and adolescents and 6%–7% of adults, impacting over 300 million people globally (Stern et al., 2020). Its high morbidity, mortality, and economic burden pose significant public health challenges worldwide (von Mutius and Smits, 2020). The primary pathogenic mechanism of asthma is typically driven by immune-inflammatory responses mediated by type 2 helper T Cells (Th2) and type 2 innate lymphoid cells (ILC2), and is regulated by type 2 cytokines such as interleukins (IL)-4, IL-5, and IL-13,and includes both allergic and non-allergic eosinophilic phenotypes (Carriera et al., 2025).

Ulcerative colitis (UC) is a chronic inflammatory bowel disease characterized by symptoms like recurrent diarrhea, bloody stools with mucus, and abdominal pain (Wang et al., 2021). Its development involves a combination of genetic factors, impaired epithelial barriers, immune system irregularities, and environmental influences (Ungaro et al., 2017). The global incidence of UC is rising, with approximately 20% of patients experiencing acute flare-ups during treatment. These flare-ups are often associated with extraintestinal symptoms, significantly affecting patients’ quality of life, social wellbeing, and mental health. Various cell types, including antigen-presenting cells (dendritic cells and macrophages), T helper cells, regulatory T Cells, and natural killer T Cells, play a crucial role in the pathogenesis of UC by modulating, inhibiting, and maintaining inflammation. Innate lymphoid cells (ILCs) may serve as key drivers in the disease mechanism, leading to numerous potential new therapeutic targets. Similarly, pro-inflammatory cytokines such as tumor necrosis factor-alpha (TNF-α), interleukins (IL)-1, IL-6, IL-9, IL-13, and IL-33 play significant roles in the pathogenesis of UC (Ungaro et al., 2017; Tatiya-Aphiradee et al., 2018).

The exact causes of asthma and ulcerative colitis (UC) remain unclear, but both share common genetic and environmental risk factors (Kisiel et al., 2023). Both diseases, as chronic inflammatory conditions, operate through distinct immune pathways, resulting in the sustained activation of inflammatory responses (Carriera et al., 2025). Emerging research highlights a link between the gut and lungs, known as the “gut-lung axis,” which plays a key role in immune regulation (Dang and Marsland, 2019). Although the mechanisms by which gut microbiota influence lung microbiota are not fully understood, there is evidence of overlapping pathological changes between intestinal and respiratory diseases, suggesting intestinal inflammation can lead to lung inflammation (Tulic et al., 2016). Studies also indicate a bidirectional relationship between gut microbiota and lung inflammation, where changes in gut microbes and their metabolites can influence respiratory disease progression *via* immune pathways (Kim et al., 2024). This interaction is linked to the higher incidence of airway conditions like asthma in patients with chronic gastrointestinal disorders. Similarly, inflammatory mediators from the lungs can impact gastrointestinal health (Marsland et al., 2015). Therefore, it has a good prospect for the specific prevention and treatment of ulcerative colitis and asthma through the intervention of intestinal flora, gut lung axis and immune inflammatory pathway (Wang et al., 2023).

Currently, sensitive and specific biomarkers for clinical use are lacking, highlighting the need to identify marker genes linked to asthma and ulcerative colitis (UC) for improved early diagnosis and treatment (Zergham et al., 2020). Recent advances in bioinformatics enabled us to retrieve gene expression data for asthma and UC from the Gene Expression Omnibus (GEO) database. We then used weighted gene co-expression network analysis (WGCNA) and machine learning to identify potential key genes shared by both diseases. Common biological pathways were explored through GO and KEGG analysis, followed by validation to ensure the reliability of hub genes. Additionally, we examined immune infiltration patterns in asthma and UC, providing valuable insights for diagnosing, tracking, and targeting therapies for these conditions.

## 2 Materials and methods

### 2.1 Microarray dataset download

We first retrieved raw gene expression data and clinical information from the GEO database (http://www.ncbi.nlm.nih.gov/geo). RNA-Seq profiles for “asthma” and “ulcerative colitis” were searched, selecting datasets with at least 15 samples per group. Ultimately, we downloaded the asthma datasets (GSE43696 and GSE63142) and UC datasets (GSE87466 and GSE92415).

### 2.2 Analysis of differentially expressed genes (DEGs)

We used the limma package in R to normalize and annotate gene expression data. Differentially expressed genes (DEGs) were identified between asthma and normal groups and between UC and normal groups, with thresholds of |log2 FC| ≥ 0.5 or one and p-adjust <0.05. The “ggplot2” “agglomerate” “dplyr” and “pheatmap” R packages were employed to generate volcano and heat maps for visualizing the DEGs.

### 2.3 Building and analyzing weighted gene Co-expression networks (WGCNA)

We used the WGCNA R package to construct co-expression networks for asthma and UC DEGs in relation to clinical characteristics. Prior to analysis, hierarchical clustering was conducted with the hclust function in R to remove outliers. The “picksoftthreshold” function in WGCNA was then used to select an optimal soft power β (ranging from 1 to 20) for network construction based on a scale-free topology. Modules were identified through topological overlap matrix analysis, with module assignments indicated by colours and module eigengenes (me). The module with the highest correlation was selected as the UC-related module. Using an online Venn diagram tool, we identified common genes by overlapping the UC-related modules.

### 2.4 Functional enrichment analysis

To explore the biological significance and pathway involvement of differentially expressed genes (DEGs), we conducted GO and KEGG enrichment analyses using the Metascape platform. The GO analysis categorizes genes into biological processes (BP), cellular components (CC), and molecular functions (MF), while KEGG focuses on pathway-level bioinformatics analysis.

### 2.5 Construction of PPI network and identification of hub genes

Using the common DEGs, a PPI network was built *via* the STRING database to analyze gene interactions, with a confidence score set above 0.4. The network was visualized in Cytoscape, and core functional genes were identified using the Maximum Clique Centrality (MCC) plugin.

### 2.6 Identification of characteristic genes and construction and verification of ROC curve

Expression data from NAFLD and HCC were used to build prediction models, including K-Nearest Neighbor (KNN), Gradient Boosting Machine (GBM), Generalized Linear Model (GLM), Decision Tree (DT), Random Forest (RF), Support Vector Machine (SVM), Least absolute shrinkage and selection operator (LASSO), Neural network (NNET), and eXtreme Gradient Boosting model (XGB). Model performance was evaluated using prediction functions, and key genes were selected by analyzing inverse cumulative distribution, residual box plots, and ROC curves.

### 2.7 Validation and construction of diagnostic model

A nomogram was constructed from cross genes identified by nine machine learning algorithms using the “RMS” package in R. The calibration curve assessed the nomogram’s predictive ability, while ROC analysis of candidate genes calculated the AUC to evaluate the prediction accuracy for asthma combined with UC. Hub gene expression levels were compared between case and control samples, and results were visualized with a block diagram.

### 2.8 Gene set enrichment analysis (GSEA) of biomarkers

To explore potential regulatory pathways and biological functions of common key DEGs in the GSE43696 and GSE87466 datasets, GSEA was conducted using the “GSEA” R package, with an adjusted p-value <0.05 as the significance threshold.

### 2.9 Analysis of immune cell infiltration

Immune cell infiltration was analyzed using the CIBERSORT deconvolution algorithm to estimate the proportions of 22 immune cells in asthma and UC samples based on immune-related gene expression. Spearman correlation was applied to assess the relationships between immune cells and key genes, with statistical significance set at p < 0.05.

### 2.10 Prediction of candidate drugs for hub genes

Based on biomarkers associated with asthma and ulcerative colitis, we utilized the DGIdb database (https://www.dgidb.org/) to predict potential therapeutic drugs. According to the interaction scores, we selected the top 10 drugs as promising candidates for disease treatment.

### 2.11 Molecular docking

3D structures of hub gene proteins (PDB format) were downloaded from the RCSB Protein Database (http://www.pdb.org/) and small molecule structures (SDF format) from PubChem (https://pubchem.ncbi.nlm.nih.gov/). The SDF files were converted to PDB format using OpenBabel. Proteins and compounds in PDB format were imported into AutoDock 1.5.7 for water removal and hydrogen addition, then saved as pdbqt files for docking. The docking affinities (kcal/mol) were calculated, with lower values indicating stronger binding. Small molecules with binding energies < −5 kcal/mol were selected for further analysis. Molecular docking results were visualized using PyMOL.

### 2.12 Molecular dynamics simulation

Based on the molecular docking results, the protein-ligand complex with the highest docking affinity was selected for molecular dynamics (MD) simulations. MD simulations were performed using Gromacs 2022 software, with the GAFF force field applied to the small molecule, and the AMBER14SB force field and TIP3P water model used for the protein. The protein and ligand files were merged to construct the simulation system for the complex. Simulations were conducted under isothermal-isobaric conditions with periodic boundary conditions. At 298 K, 100 ps NVT and NPT equilibration simulations were first carried out, followed by a 100 ns MD simulation with conformations saved every 10 ps? After the simulation, the trajectories were analyzed using VMD and PyMOL.

## 3 Results

### 3.1 Identification of differential genes

**FIGURE 1:**
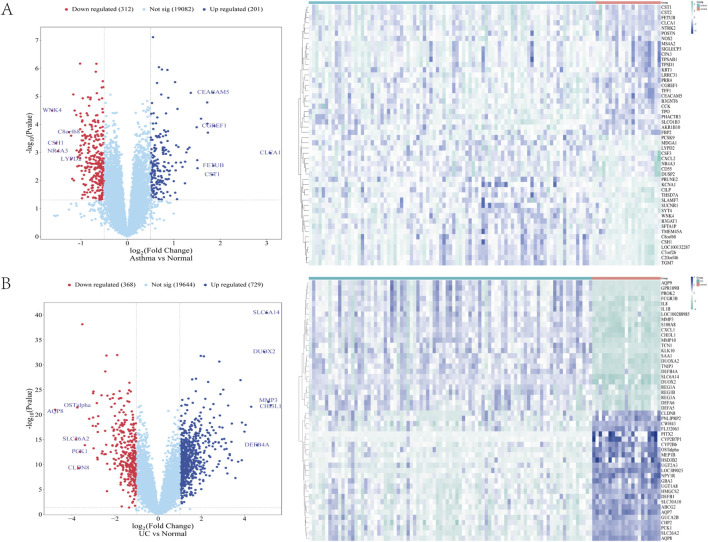
**(A, B)** The heat map and volcano map of differentially expressed genes in GSE43696 and GSE87466 datasets, respectively **(A)** Expression characteristics of differentially expressed genes (DEGs) in patients with asthma **(B)** Expression characteristics of differentially expressed genes (DEGs) in patients with ulcerative colitis (UC). In the DEGs Heatmap, green and blue indicate mRNA upregulation and downregulation, respectively. The darker the colour, the higher the expression level. Normal is a healthy control group, and asthma and UC are disease groups.

In the GSE43696 asthma dataset, 513 DEGs were identified, including 201 upregulated and 312 downregulated genes. In the GSE87466 UC dataset, 1,097 DEGs were found, with 729 upregulated and 368 downregulated genes. The DEGs from both datasets were visualized using volcano plots and heat maps (Figures 1A, B) (Supplementary Table S1).

### 3.2 WGCNA identifies key module genes in UC samples

**FIGURE 2:**
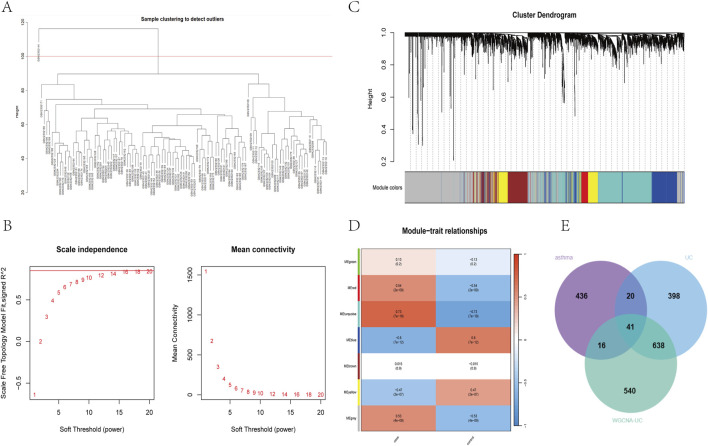
Construction of UC weighted gene coexpression network **(A)** clustering dendrogram of samples **(B)** Determine the soft threshold power (β), including the scale-free fitting index of various soft threshold powers (left) and the average connectivity of various soft threshold powers (right) **(C)** The dendrogram and seven modules of all DEGs clustered based on the dissimilarity measure (1-tom) are displayed in corresponding colours **(D)** Correlation coefficient between gene module and UC occurrence **(E)** Venn diagram of 41 cross genes of UC-related genes identified by overlapping DEGs and WGCNA.

To identify key genes associated with the UC phenotype, we constructed a gene co-expression network using the WGCNA algorithm. After removing abnormal samples, we generated a clustering dendrogram for UC and control groups (Figure 2A). A soft threshold of 16 (*R* ^2^ = 0.9) was chosen to build a scale-free network (Figure 2B). We merged modules based on a cut-off value, resulting in seven co-expression modules (Figure 2C). The “Turquoise” module, containing 1,235 genes, showed the strongest clinical relevance to SAP (COR = 0.73, P = 7 × 10^−9^) and was identified as the most valuable module in relation to UC phenotype (Figure 2D). Relevant shared genes were obtained by crossing the UC module genes obtained by WGCNA with 513 DEGs obtained from the gse43696 dataset of asthma and 1,097 DEGs obtained from the gse87466 dataset of UC, as shown in the Venn diagram in Figure 2E (Supplementary Table S2).

### 3.3 Functional enrichment analysis

**FIGURE 3:**
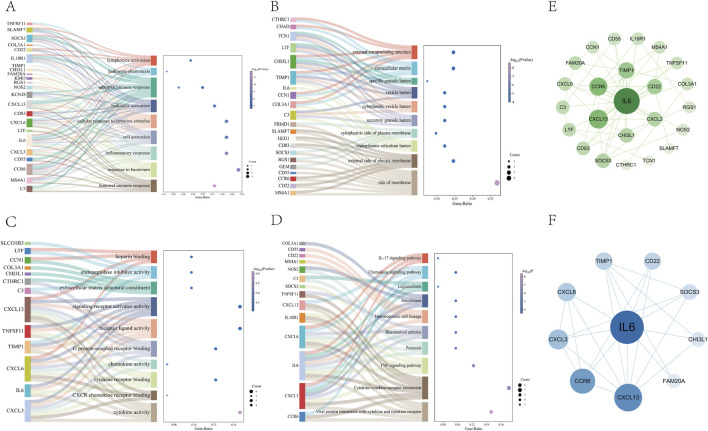
Go and KEGG pathway enrichment analysis of DEGs **(A–C)** The 10 most significant of go analysis. BP, CC and MF **(D)** The 10 most significant of KEGG analysis **(E)** The PPI network consists of cross-targets of 24 genes **(F)** The cytohubba plugin was used to access the 10 genes with the most significant associations.

We conducted GO biological process enrichment and KEGG pathway analysis to identify common regulatory mechanisms between asthma and UC. Using the Metascape platform, we performed 232 GO and 15 KEGG enrichment analyses. This revealed 195 BPs, 23 MFs, and 14 CCs through GO enrichment. In Go analysis, most DEGs are mainly involved in human immune response inflammatory response(BP); side of membrane、external side of plasma membrane(CC); Cytokine activity, CXCR chemokine receptor binding (MF) (Figures 3A–C). KEGG enrichment analysis showed that most DEGs were mainly enriched in cytokine receptor interaction, TNF signaling pathway, chemokine signaling pathway and IL-17 signaling pathway (Figure 3D).

### 3.4 PPI network construction and hub gene identification

To explore protein-protein interactions and identify hub genes in asthma and UC, we constructed a PPI network using the STRING database. After removing isolated nodes, the network contained 24 nodes and 55 edges (Figure 3E). Using the MCC plugin in Cytoscape, we identified the core functional genes: IL6, CXCL13, CCR6, CXCL3, CXCL6, TIMP1, CD22, SOCS3, CHI3L1, and FAM20A (Figure 3F) (Supplementary Table S3).

### 3.5 Selection and validation results of machine learning models

**FIGURE 4:**
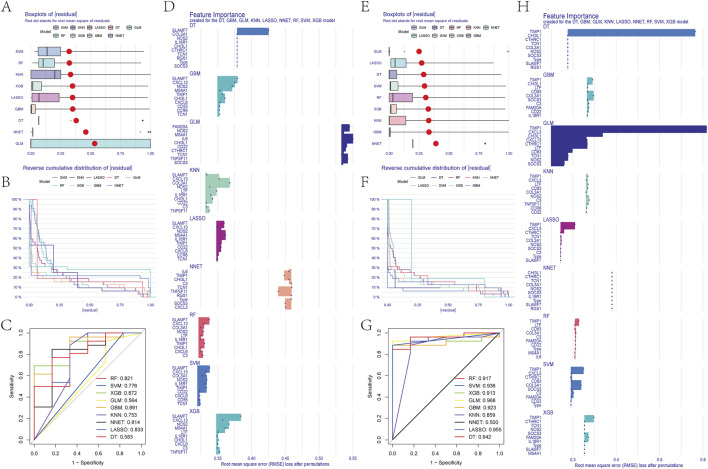
**(A)** Block diagram of nine machine learning model residuals in asthma **(B)** Inverse cumulative distribution of residuals of nine machine learning models in asthma **(C)** ROC of nine machine learning models in asthma **(D)** Feature importance histogram of nine machine learning models in asthma **(E)** Block diagram of nine machine learning model residuals in ulcerative colitis **(F)** Inverse cumulative distribution of nine machine learning model residuals in ulcerative colitis **(G)** ROC of nine machine learning models in ulcerative colitis **(H)** Feature importance histogram of nine machine learning models in ulcerative colitis.

**FIGURE 5:**
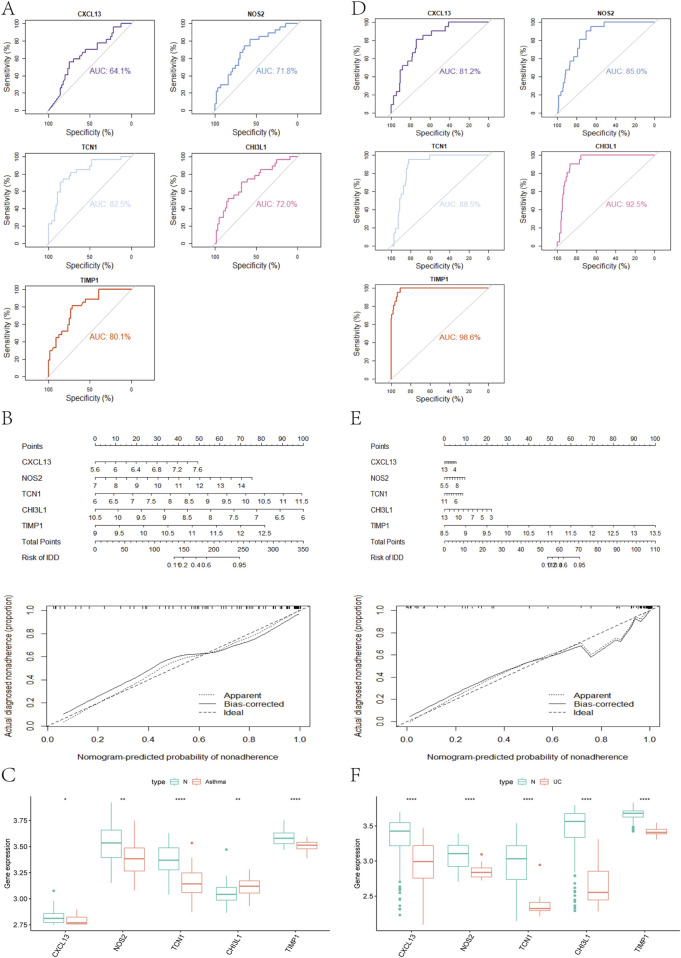
**(A)** Analysis of hub genes in the GSE63142 dataset using the ROC curve **(B)** Nomogram and a calibration curve of five biomarkers of asthma **(C)** Expression of asthma-related hub genes in the GSE63142 dataset **(D)** The hub genes in the GSE92415 dataset were analyzed using ROC curves **(E)** Nomogram and a calibration curve of five biomarkers of UC **(F)** Expression of UC-related hub genes in the GSE63142 dataset.

We used the DEGs data of asthma and UC to construct the results of SVM, RF, XGB, KNN, LASSO, NNET, DT, GBM, and GLM machine learning prediction models respectively. In the asthma model, according to the residual box diagram, inverse cumulative distribution diagram and ROC curve (Figures 4A–C), GBM method has the highest area under the ROC curve, the lowest residual value and the lowest inverse cumulative value. Therefore, the GBM method was considered to be the most accurate and was selected as the best model for further analysis. Similarly, the GLM method is considered to be the most accurate method in the UC model (Figures 4E–G). GBM and GLM models provided importance scores for the selected signature genes, as shown in Figures 4D, H, revealing 10 signature genes. Nomograms were constructed using the 10 genes with the highest importance scores. Then, the genes selected by the two methods were crossed, and finally, the key genes involved in the progression of asthma to UC (CXCL13, NOS2, TCN1, CHI3L1 and TIMP1) were obtained. To validate the efficacy of core hub genes, we validated five candidate hub genes in two other validation datasets (GSE63142 for asthma and GSE92415 for UC). The results showed that only four genes (NOS2, TCN1, CHI3L1, TIMP1) showed statistically significant differences in expression in the data sets validated by asthma and UC compared with the control group (Figures 5A, D). In order to enhance clinical utility, nomograms containing five biomarkers were generated to predict the progression of asthma and UC, respectively (Figures 5B, E). The calibration curve showed that the difference between the actual value and the predicted value of risk was small, which indicated that the nomogram had a high diagnostic value. In the validation set, five biomarkers showed significant differences (Figures 5C, F) (Supplementary Table S4).

### 3.6 GSEA analysis

**FIGURE 6:**
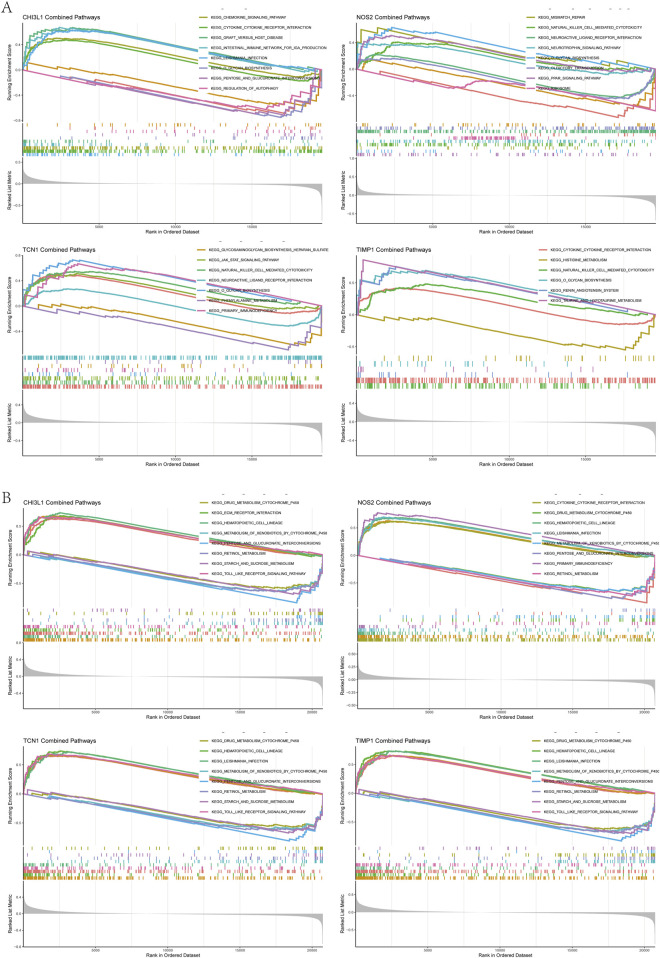
Gene set enrichment analysis **(A)** GSEA results of chi3l1, NOS2, TCN1, TIMP1 in the asthma dataset **(B)** GSEA results of chi3l1, NOS2, TCN1, and TIMP1 in the UC dataset.

To better understand the impact of biomarkers on asthma and UC progression, we performed GSEA. In asthma, significant enrichment was observed in the cytokine receptor interaction, chemokine signaling, NK cell-mediated cytotoxicity, JAK-STAT, and Toll-like receptor pathways (Figure 6A). In UC, significant enrichment was found in the cytokine receptor interaction, chemokine signaling, Fc epsilon RI, RIG-I-like receptor, NOD-like receptor, and leukocyte transendothelial migration pathways, compared to controls (Figure 6B). Notably, immune pathways like cytokine receptor interaction and chemokine signaling are shared between asthma and UC (Supplementary Table S5).

### 3.7 Analysis of immune cell infiltration

**FIGURE 7:**
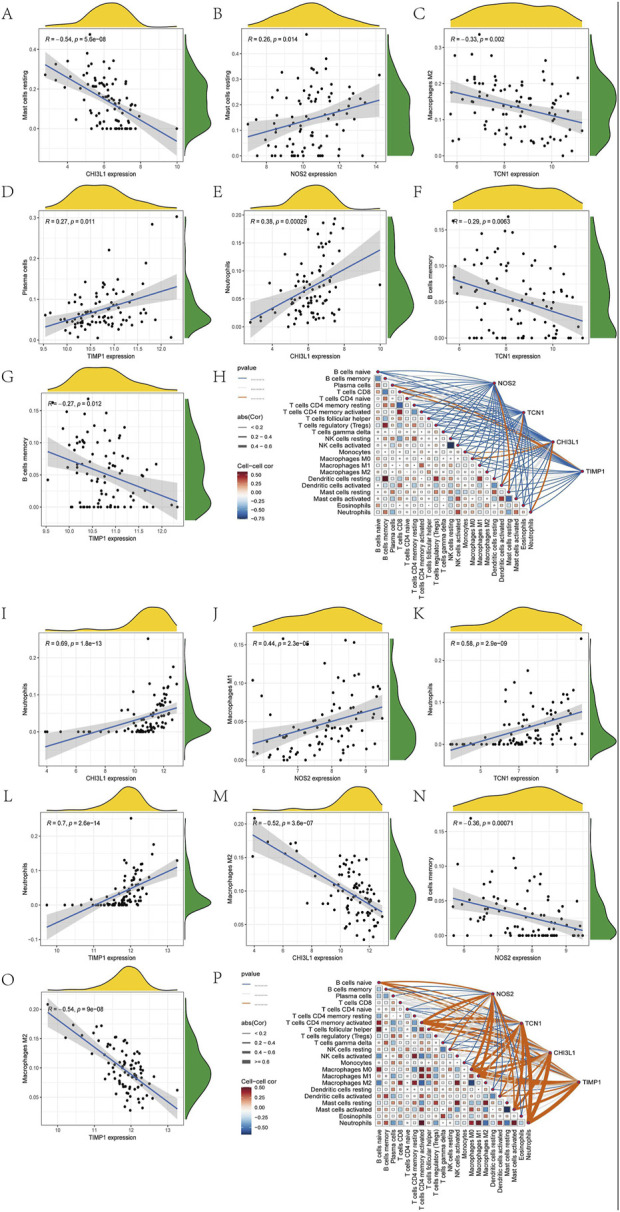
Immune cell infiltration analysis. Ashma data correlation analysis **(A)** Correlation analysis between the expression of CHI3L1 and Mast cells resting **(B)** Correlation analysis between NOS2 expression and Mast cells resting **(C)** Correlation analysis of TCN1 expression and Macrophages M2 **(D)** Correlation analysis between TIMP1 expression and Plasma cells **(E)** Correlation analysis between the expression of CHI3L1 and Neutrophils **(F)** Correlation analysis of TCN1 expression and B Cells memory **(G)** Correlation analysis between TIMP1 expression and B Cells memory **(H)** The correlation diagram of the association between immune cells and NOS2, TCN1, CHI3L1, and TIMP1 in asthma. Correlation analysis of UC data **(I)** Correlation analysis of CHI3L1 expression and Neutrophils **(J)** Correlation analysis between NOS2 expression and Macrophages M1 **(K)** Correlation analysis between the expression of TCN1 and Neutrophils (Harb et al., 2020). **(L)** Correlation analysis of TIMP1 expression and Neutrophils. **(M)** Correlation analysis between the expression of CHI3L1 and Macrophages M2 **(N)** Correlation analysis between NOS2 expression and B Cells memory **(O)** Correlation analysis of TIMP1 expression and Macrophages M2 **(P)** Correlation diagram of the association of immune cells with NOS2, TCN1, CHI3L1, and TIMP1 in UC.

Since the intersecting genes of asthma and UC are primarily involved in inflammation and immune regulation, we used the CIBERSORT algorithm to analyze immune cell infiltration in the AMI and IHF datasets. The immune cells and biomarkers with significant correlation were screened by R-value (p < 0.05). Asthma data results show that NOS2 is resting with Mast cells (R = 0.26, p = 0.014), TIMP1 is resting with Plasma cells (R = 0.27, p = 0.011), CHI3L1 is resting with Neutrophils (R = 0.38, p = 0.00029) is positively correlated (Figures 7B, D, E). CHI3L1 is associated with Mast cells resting (R = −0.54, p = 5.6e-08), TCN1 and Macrophages M2 (R = −0.33, p = 0.002), TCN1 and B Cells memory (R = −0.29, p = 0.0063), TIMP1 were negatively correlated with B Cells memory (R = −0.27, p = 0.012) (Figures 7A, C, F, G). The UC data results show that CHI3L1 is associated with Neutrophils (R = 0.69, p = 1.8e-13), NOS2 and Macrophages M1 (R = 0.44, p = 2.3e-05), TCN1 and Neutrophils (R = 0.58, p = 2.9e-09), TIMP1 were positively correlated with Neutrophils (R = 0.7, p = 2.6e-14) (Figures 7I–L). CHI3L1 and Macrophages M2 (R = −0.52, p = 3.6e-07), NOS2 and B Cells memory (R = −0.36, p = 0.00071), TIMP1 and Macrophages M2 (R = −0.54, p = 9e-08) showed a negative correlation (Figures 7M–O). Asthma results showed (Figure 7H) that the expression of CHI3L1 was positively correlated with Monocytes, Macrophages M0 and Neutrophils, while on the contrary, CHI3L1 was negatively correlated with Plasma cells and Mast cells resting. The expression of NOS2 was positively correlated with Mast cells resting and negatively correlated with T Cells CD4 memory resting and B Cells memory. The expression of TCN1 was positively correlated with Dendritic cells activated and negatively correlated with Macrophages M2, Dendritic cells resting, B Cells memory and T Cells CD4 naive. TIMP1 was negatively correlated with Plasma cells and T Cells CD8, B Cells memory, T Cells CD4 naive, Macrophages M2 and Dendritic cells resting. The results of UC (Figure 7P) showed that the expression of CHI3L1 was positively correlated with Neutrophils and Macrophages M0 and negatively correlated with Mast cells resting and Macrophages M2. The expression of NOS2 was positively correlated with T Cells CD4 memory activated and Macrophages M1, and negatively correlated with T Cells regulatory (Tregs) and NK cells activated. The expression of TCN1 was positively correlated with T Cells CD4 memory activated and Neutrophils and negatively correlated with Mast cells resting and Macrophages M2. TIMP1 is positively correlated with Macrophages M0 and Neutrophils. It is negatively correlated with Mast cells resting and Macrophages M2. In conclusion, Asthma and UC patients have varying degrees of multiple immune cell infiltrations that may be potential regulatory points for treatment (Supplementary Table S6).

### 3.8 Candidate drug prediction

This study employed the DGIdb database to predict potential intervention drugs. Table 1 presents the top 10 compounds based on adjusted p-values. PD 98059 (PD 98059 CTD 00003206), beclomethasone (beclomethasone CTD 00005468), and isoproterenol (isoproterenol CTD 00006175) are three important drugs associated with key targets.

**TABLE 1:** Candidate drug predicted using DGIdb.

Drug names	P-value	Adjusted P-value	Genes
PD 98059 CTD 00003206	8.80E-06	0.001600693	NOS2; CHI3L1; TIMP1
2,4-Diisocyanato-1-methylbenzene CTD 00006908	1.39E-05	0.001600693	NOS2; TIMP1
DIALLYL DISULFIDE CTD 00001321	2.58E-05	0.001600693	NOS2; TIMP1
Gadodiamide hydrate CTD 00002623	2.70E-05	0.001600693	NOS2; TIMP1
beclomethasone CTD 00005468	6.60E-05	0.00313045	NOS2; TIMP1
isoproterenol CTD 00006175	9.20E-05	0.003632299	NOS2; TIMP1
chitosamine CTD 00006030	1.09E-04	0.003691714	NOS2; CHI3L1
Diallyl trisulfide CTD 00001934	1.25E-04	0.003698062	NOS2; TIMP1
Dinoprostone CTD 00007049	2.38E-04	0.006268338	NOS2; TIMP1
Isotretinoin HL60 UP	2.73E-04	0.006468926	CHI3L1; TIMP1

### 3.9 Molecular docking

**FIGURE 8:**
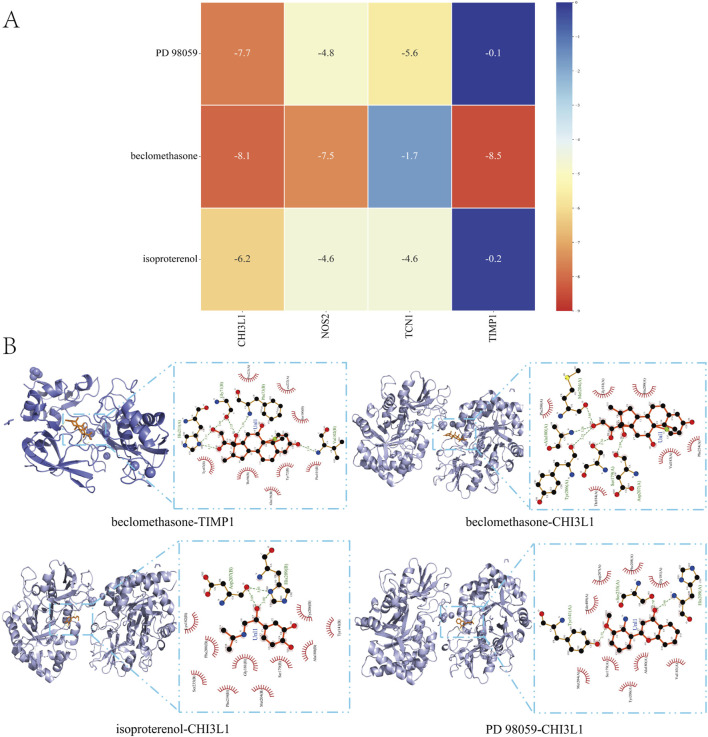
Molecular Docking **(A)** Matrix Heatmap of docking binding energy: the redder the colour, the more important the node is **(B)** Docking results of available protein-small molecules. Beclomethasone docked TIMP1 and CHI3L1, isoproterenol docked CHI3L1, and PD 98059 docked CHI3L1.

To assess the affinity of candidate drugs for their targets and predict their therapeutic potential for asthma and UC, we conducted molecular docking. The drugs PD 98059, beclomethasone and isoproterenol were respectively docked with the core targets NOS2, TCN1, CHI3L1 and TIMP1. Using AutoDock Vina v.1.5.7, we identified binding sites and interactions, generating binding energies for each drug-target interaction (Figures 8A, B). The drugs interacted with their protein targets via hydrogen bonds and electrostatic forces. Beclomethasone showed the lowest binding energy with TIMP1 (−8.5 kcal/mol), indicating a highly stable binding. The detailed information of the molecular docking results mentioned above can be found in Supplementary Table S7.

### 3.10 Molecular dynamics simulation

**FIGURE 9:**
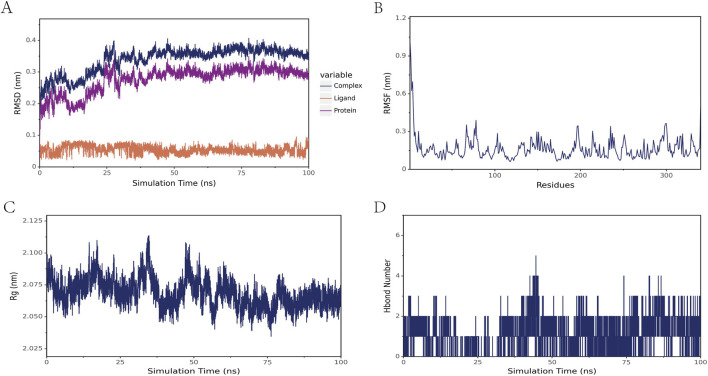
Molecular dynamics simulation results of the TIMP1-Beclomethasone complex **(A)** RMSD of the complex, protein, and small-molecule ligand **(B)** RMSF of the protein within the complex **(C)** Radius of Gyration (Rg) of the complex **(D)** Number of hydrogen bonds (Hbond number) in the complex.

In this study, molecular dynamics simulations were performed to further confirm the stability of the ligand-receptor binding, specifically to verify the binding affinity between TIMP1 and Beclomethasone. RMSD (Root Mean Square Deviation) is an indicator used to assess the stability of protein-ligand complexes. The smaller the RMSD, the less the overall structural change of the complex, indicating greater stability. Figure 9A illustrates the RMSD values of the three systems over time. At 30 ns, all systems reached a stable state: the TIMP1-Beclomethasone complex maintained an RMSD of 0.35 nm, and from 30 to 100 ns, the RMSD remained stable with minimal fluctuation, suggesting a very stable binding between TIMP1 and Beclomethasone, and a strong binding state. The fluctuation of amino acid residues in the protein after small molecule binding is reflected by RMSF (Root Mean Square Fluctuation). The results (Figure 9B) indicate minimal conformational changes in the amino acids during the simulation process. Rg (Radius of Gyration) analysis was employed to characterize the compactness and stability of the structure during the dynamic simulation. This parameter reflects the distribution of molecular atoms relative to the center of mass and serves as an important measure of the overall compactness of the protein-small molecule complex. As shown in Figure 9C, the Rg of the complex ranged from 2.050 to 2.100, indicating that the complex is structurally stable and compact. To investigate the hydrogen bond characteristics at the binding site, the number of hydrogen bonds between the ligand and the protein was calculated. Figure 9D shows that the number of hydrogen bonds between the small molecule and the protein fluctuated mainly between 0 and 2.

## 4 Discussion

Recently, bioinformatics and machine learning have been widely used to identify key genes, biomarkers, signaling pathways, and therapeutic targets, aiding in the comprehensive understanding of diseases (Huang et al., 2024). Asthma and ulcerative colitis are chronic immune-mediated inflammatory diseases of the respiratory and gastrointestinal systems, respectively. Over recent decades, their incidence and prevalence have risen globally, placing a significant burden on individuals, populations, and healthcare systems (Kuenzig et al., 2017). The potential link between these two conditions has garnered increasing attention (Zou et al., 2023).

Functional enrichment analysis revealed that the key genes of Asthma-UC are primarily involved in immune response, inflammation, and cytokine activity. These genes are associated with pathways such as cytokine receptor interaction, TNF signaling, chemokine signaling, and IL-17 signaling.

TNF-α is crucial in the pathogenesis of asthma and may serve as a candidate gene for the condition (Huang et al., 2014), with its overproduction contributing to acute and chronic inflammation, including asthma (Richmond et al., 2015). In addition, immune cells produce proinflammatory cytokines, which play a crucial role in the pathogenesis of UC. Among them, cytokines such as TNF serve as key immune mediators, contributing to persistent immune dysregulation and intestinal inflammation (Kobayashi et al., 2020; Suzuki et al., 2014). TNF-α, produced by immune cells in the gut of UC patients, is linked to Th17 differentiation and regulates innate and adaptive immunity (Nakase et al., 2022). Over recent decades, TNF-α monoclonal antibodies, like infliximab, have shown therapeutic effectiveness in UC treatment (Guo and Wang, 2023).

IL-17E (IL-25) is key to regulating type 2 immune responses and driving inflammatory conditions like allergic asthma through IL-17RA and IL-17RB receptors (Wilson et al., 2022). Research highlights IL-17A’s pivotal role in severe asthma, with elevated IL-17 levels observed in sputum and bronchial biopsies from such patients (Manni et al., 2014; Chesné et al., 2014). In a murine asthma model, NF-κB activation in epithelial cells was linked to increased neutrophilia, eosinophilia, and IL-17 production (Pantano et al., 2008). Novielo et al. highlighted the critical role of the IL-23/IL-17 axis in UC pathogenesis (Noviello et al., 2021). Monoclonal antibody treatments targeting IL-17A have been shown to trigger or worsen UC, highlighting IL-17’s role in preserving intestinal homeostasis and regulating innate inflammation (Fauny et al., 2020). Notably, IL-17 cytokines are upregulated at inflammation sites and synergize with TNF-α to amplify the inflammatory response (Camargo et al., 2023).

In our study, 10 biomarkers (IL6, CXCL13, CCR6,CXCL3, CXCL6, TIMP1, CD22, SOCS3, CHI3L1, and FAM20A) were identified as the top ranked hub targets. IL-6 functions as both a pro-inflammatory marker and an active contributor to asthma pathogenesis, potentially driving lung function decline in untreated patients (Rincon and Irvin, 2012). Elevated intestinal levels of the inflammatory cytokine IL-6 are positively correlated with the activity and severity of ulcerative colitis (Cui et al., 2023). CXCL13 is a potent B Cell chemokine that is released in high concentrations in the airways of asthmatics and plays an important role in allergic inflammation (Baay-Guzman et al., 2012). Targeting CXCL13 + T Cells or CXCL13 itself may help reduce the production of pathogenic B Cells, thereby alleviating colonic inflammation (Uzzan et al., 2022). Cxcl3 is a well-known potent neutrophil chemokine and a potent mediator of smooth muscle cell migration in normal and asthmatic airways (Al-Alwan et al., 2013). Relevant experiments showed that the expression of CXCL3 in colon tissue of UC rats was elevated 28-fold, which may play a key role in the pathogenesis of inflammation-based UC (Boshagh et al., 2019).

GSEA analysis showed that NOS2, TCN1, CHI3L1 and TIMP1 were jointly involved in cytokine receptor interaction in asthma and ulcerative colitis chemokine signaling pathway. CCL6 deficiency significantly reduces ovalbumin-induced airway eosinophilia, mucus hypersecretion, and Th2 responses, with the CCL6-CCR1 axis serving as a key regulator in asthma pathogenesis and a promising therapeutic target (Du et al., 2021). Additionally, CCL6 plays a crucial role in immune cell recruitment and intestinal inflammation (Cao et al., 2016). Similarly, CXCL8, a well-studied proinflammatory chemokine, is linked to inflammation-driven diseases like asthma and inflammatory bowel disease through its dysregulated signaling (Ha et al., 2017).

Heparanase (HPSE) is an essential enzyme that degrades the extracellular matrix (ECM) and basement membrane (BM), playing a crucial role in infectious and autoimmune inflammatory processes (Jin and Cui, 2018). HPSE promotes the recruitment of eosinophils to the lungs in response to allergens by degrading heparan sulfate and releasing cytokines bound to it. Relevant experiments have shown that pharmacological inhibition of heparanase suppresses bronchial hyperresponsiveness (BHR) (Morris et al., 2015). Studies have also observed that the deficiency of HPSE reduces dendritic cell numbers in the lungs and selectively eliminates Th2 cell-mediated immune responses that induce asthma, providing potential new therapeutic targets for anti-inflammatory drugs to treat asthma and other allergic diseases (Poon et al., 2014). During chronic inflammation in the colon, such as in UC, HPSE degrades heparan sulfate (HS) chains, disrupting the integrity of the ECM and BM, thus promoting the directional migration of inflammatory cells and releasing bioactive factors from the ECM and cell surfaces. Additionally, HPSE can mediate the release of cytokines such as TNF-α and IL-6 from macrophages, exacerbating intestinal inflammation, inducing tumorigenesis, and promoting angiogenesis (Waterman et al., 2007; Lerner et al., 2011; Hermano et al., 2012; Cui et al., 2021).

LRP8, through its influence on the Wnt signaling pathway (Fang et al., 2022), may contribute to the pathogenesis of asthma, including airway inflammation and remodeling, by interacting with specific Wnt ligands in various cell types. The Wnt signaling pathway is involved in a range of physiological and pathological processes, including neuroinflammation and autoimmune diseases, and its regulation could impact disease progression (Baarsma and Königshoff, 2017).

Notch4 disrupts Treg cells into TH2 and TH17 effector T (Teff) cells through Wnt and Hippo pathway-dependent mechanisms, thereby enhancing immune cell infiltration in the airways and exacerbating the inflammatory response in asthma. As a result, related studies have identified Notch4-mediated disruption of immune tolerance as a fundamental mechanism of tissue inflammation in asthma (Harb et al., 2020). The discovery of a novel Notch4-Wnt-GDF15 axis in controlling allergic asthma in mice, and its subsequent validation in patients with severe asthma, undoubtedly offers new therapeutic prospects for restoring pulmonary immune tolerance and maintaining systemic balance (Hammad and Lambrecht, 2020). Notch signaling, which plays a critical role in maintaining the proliferation and differentiation of colon epithelial cells, has been linked to severe inflammation and colitis when chronic inhibition of the Notch pathway, combined with intestinal infection, leads to changes in mucosal components, bacterial dysbiosis, and loss of tight junction integrity (Ahmed et al., 2018). Relevant animal studies provide compelling evidence of Notch signaling’s involvement in the crosstalk between innate and adaptive immune cells (Cahill et al., 2015), and demonstrate a significant role for Notch4 in embryonic dermal lymphangiogenesis (Muley et al., 2022).

In addition, we identified NOS2, TCN1, CHI3L1, and TIMP1 as possible diagnostic markers for UC and asthma. NOS2 (inducible nitric oxide synthase) plays a critical role in immune-inflammatory diseases (Kanwar et al., 2009). Present in the respiratory epithelium, NOS2 is strongly linked to exhaled NO levels in asthmatic children and may serve as a redox-related marker of asthma progression (Salam et al., 2011; Qi et al., 2018). Increased iNOS activity and expression have been observed in the colonic mucosa of UC patients, with NOS2 inhibition shown to alleviate intestinal inflammation, highlighting its role in UC pathogenesis (Zhang et al., 2022; Bernstein et al., 2007).

TCN1 is linked to various immune checkpoint markers and immune cells, playing key roles in cell metabolism and proliferation (Li H. et al., 2022). Its expression is elevated in the sputum of asthmatic patients, correlating positively with inflammatory markers and negatively with lung function, making it a potential biomarker for asthma diagnosis and treatment (Xu et al., 2023). While TCN1 expression differences have been noted in other studies, current evidence does not support its use as a UC biomarker (Cheng et al., 2020; Hu et al., 2022). However, elevated TCN1 levels have been observed in refractory UC cases (Komeda et al., 2020). In our study, TCN1 showed excellent discrimination ability, which indicated that TCN1 was expected to be a candidate biomarker for asthma and UC.

CHI3L1, part of the glycoside hydrolase family 18, plays a key role in tissue injury, inflammation, repair, and remodeling. Elevated CHI3L1 levels have been detected in the serum and lungs of asthma patients (Zhao et al., 2020). The promoter SNP (−131C→G) in CHI3L1 is linked to higher serum CHI3L1 levels, asthma susceptibility, bronchial hyperresponsiveness, and lung function indicators (Chupp et al., 2007; James et al., 2016; Tang et al., 2010). In inflammatory bowel disease, CHI3L1 aggravates inflammation and promotes bacterial adhesion and invasion by interacting with bacterial chitin-binding proteins (Buisson et al., 2016). Targeting CHI3L1 activity offers a promising therapeutic strategy.

TIMP1, a metalloproteinase inhibitor, forms irreversible complexes with MMPs like MMP10 and MMP13, suppressing protease activity and limiting collagen degradation (Li Y. et al., 2022). Studies suggest TIMP1 enhances eosinophilic airway inflammation, airway remodeling, and lung function decline in severe asthma, making it a potential marker for predicting persistent eosinophilic inflammation and poor outcomes in severe asthma (Cao et al., 2023). In UC patients, TIMP1 expression is elevated and associated with innate and B cell-mediated immune responses, immune regulation, and T Cell activation, playing an immunomodulatory role in UC pathogenesis (Pan et al., 2023). TIMP1 also influences the prognosis of IBD-associated CRC and may serve as a marker for monitoring intestinal mucosal healing in UC (Li Y. et al., 2022; Altadill et al., 2021).

In conclusion, NOS2, TCN1, CHI3L1 and TIMP1, as key immune and inflammatory regulatory targets, play an important role in the comorbidity of asthma and ulcerative colitis. The functions of these targets are not only of great significance in the pathogenesis of a single disease, but their interactions in comorbidities may promote the dysregulation of systemic immune responses, leading to the exacerbation of patients’ conditions. Therefore, the combined treatment strategies targeting these targets may provide more effective treatment options for patients with comorbid asthma and ulcerative colitis.

CIBERSORT analysis revealed significant differences in immune cell infiltration between asthma, UC, and the control group, with several immune cell subtypes closely linked to the biological processes of both diseases. Recent studies confirm that immune cell infiltration plays a critical role in the development of asthma and UC. Our analysis found a strong correlation between the expression of hub genes (NOS2, TCN1, CHI3L1, and TIMP1) and immune cell infiltration in disease comorbidities. Specifically, these genes were significantly associated with Macrophage M1, Macrophage M2, resting mast cells, memory B Cells, and resting memory CD4 T Cells.

Macrophages, the most abundant immune cells in the lungs, play a crucial role in asthma. They differentiate into pro-inflammatory M1 or anti-inflammatory M2 phenotypes based on local stimuli. M1 macrophages are predominant in non-allergic asthma, while M2 macrophages are more involved in allergic asthma (Saradna et al., 2018). M1 macrophages are particularly linked to severe asthma, especially in patients unresponsive to systemic corticosteroids (Oriss et al., 2017). Similarly, in the gastrointestinal mucosa, macrophages are essential for intestinal homeostasis, with M1 macrophages promoting inflammation and mucosal damage, while M2 macrophages aid in tissue repair and reduce inflammation, thus alleviating IBD symptoms (Bain and Mowat, 2014; Huo and Wang, 2023).

Mast cells (MCs) are versatile immune effectors widely distributed throughout the body (Paivandy and Pejler, 2021). They play a central role in asthma pathogenesis by producing mediators that regulate both innate and adaptive immunity in the lungs. Mast cell activation, triggered by allergic and non-allergic stimuli, is crucial for initiating and maintaining the allergic inflammatory cycle, primarily through the secretion of type 2 (Th2) cytokines. Key triggers for mast cell activation in asthma include allergen-stimulated IgE receptors (FCεRI), toll-like receptors, and cytokines that activate alarmin receptors (e.g., TSLP, IL-33) (Bradding and Arthur, 2016). In ulcerative colitis, a reduction in quiescent mast cells in affected tissues compared to normal tissue has also been observed (Ding et al., 2023). Our findings showed a strong correlation between the expression of four diagnostic markers and immune cell infiltration, highlighting the critical role of immune mechanisms in the inflammation and immune responses of asthma complicated by UC.

In addition, we predict the progression of potentially effective therapeutics for UC with asthma. We obtained 10 overlapping potential drugs targeting NOS2, TCN1, CHI3L1 and TIMP1 from the DGIdb database and verified the molecular docking of three of them. Among them, PD 98059, an ERK inhibitor, acts as a downstream regulator of Kv1.3 channel inhibitors in neutrophilic asthma and has therapeutic potential for the treatment of asthma (Zhou et al., 2018). It is well known that beclomethasone is widely used in the treatment of asthma. Similarly, in Italy and a few other European and non-European countries, oral controlled-release BDP preparations have been approved for the treatment of active mild to moderate ulcerative colitis (Manguso et al., 2016). Relevant studies have shown that continuous inhalation of low-dose l-isoproterenol in the emergency department and hospital environment is superior to salbutamol in the treatment of pediatric patients with acute severe attacks of asthma. Compared with salbutamol, it has a faster onset of action and fewer adverse events (Katsunuma et al., 2019). MD simulations are crucial for understanding protein conformational changes and dynamic mechanisms, and are commonly used in drug design and target validation. The results above indicate that the binding between TIMP1 and Beclomethasone is highly stable. Therefore, our findings may provide valuable insights for developing effective treatments for asthma and ulcerative colitis UC.

This study identified potential diagnostic markers and molecular mechanisms of asthma and ulcerative colitis using bioinformatics and machine learning. However, a key limitation is the lack of experimental validation, which is essential to confirm the biological relevance of our findings. In the future, our research will focus on *in vitro* and *in vivo* experiments to further validate the identified biomarkers. Despite this limitation, our study provides a valuable computational framework for future research and potential clinical applications.

## 5 Conclusion

In conclusion, our bioinformatics analysis identified NOS2, TCN1, CHI3L1 and TIMP1 as potential diagnostic biomarkers for asthma and UC. These genes are likely involved in disease pathogenesis through their roles in immunity and inflammation. Furthermore, immune infiltrating cells, such as Macrophage M1, Macrophage M2, resting mast cells, memory B Cells, and resting memory CD4 T Cells, dominate in both diseases. These findings highlight the key role of immune responses in asthma and UC, driven by interactions between hub genes and immune cells. These biomarkers offer new insights for personalized diagnosis, prevention, and treatment of asthma and UC and could advance our understanding of the gut-lung axis.

## Data Availability

The datasets presented in this study can be found in online repositories. The names of the repository/repositories and accession number(s) can be found in the article/Supplementary Material.

## Abbreviations

KEGG, Kyoto Encyclopedia of Genes and Genomes; UC, Ulcerative colitis; GEO, Gene Expression Omnibus; GO, Gene Ontology; WGCNA, Weighted gene co-expression network analysis; PPI, protein protein interaction; KNN, K-Nearest Neighbor; GBM, Grandient Boosting Machine; GLM, Generalized Linear Models; DT, Decision Tree; RF, Random Forest model; SVM, Support Vector Machine; XGB, eXtreme Gradient Boosting model; ROC, receiver operating characteristic; LASSO, Least absolute shrinkage and selection operator; NNET, Neural network.
